# Comparison of EI-GC-MS/MS, APCI-LC-MS/MS, and ESI-LC-MS/MS for the Simultaneous Analysis of Nine Nitrosamines Eluted from Synthetic Resins into Artificial Saliva and Health Risk Assessment

**DOI:** 10.3390/toxics9100230

**Published:** 2021-09-23

**Authors:** Hyungsoo Kim, Daekwan Sung, Honghyeon Yu, Daeyong Jang, Yeji Koo, Seungha Lee, Kyungmin Lim, Dalwoong Choi

**Affiliations:** 1 Research Institute of Health Sciences, College of Health Science, Korea University, Seoul 02841, Korea; dkss9900@naver.com (H.K.); foxrice2@naver.com (D.J.); 5270463@hanmail.net (Y.K.); km1004sh10@gmail.com (S.L.); 2 Transdisciplinary Major in Learning Health Systems, Department of Health and Safety Convergence Science, Graduate School, Korea University, Seoul 02841, Korea; 3 Department of Public Health Science, Graduate School, Korea University, Seoul 02841, Korea; dagoan@naver.com (D.S.); hyuneee10@daum.net (H.Y.); 4 College of Pharmacy, Ewha Womans University, Seoul 03670, Korea

**Keywords:** nitrosamines, gas chromatography-tandem mass spectrometry, liquid chromatography-tandem mass spectrometry, method validation, risk assessment

## Abstract

Nitrosamines can be produced during the manufacture of rubber-type products such as pacifiers or the nipples of baby bottles. Humans can be exposed to the nitrosamines in these products when they are eluted into saliva. In this study, we compared the efficiency of electron impact ionization (EI), atmospheric pressure chemical ionization (APCI), and electrospray ionization (ESI) methods for the analysis of nine nitrosamines eluted into artificial saliva. In addition, nine nitrosamines eluted from 54 rubber-type products (rubber, thermoplastic elastomer, thermoplastic polyurethane, and polyurethane) marketed in Korea were monitored. Finally, non-carcinogenic and carcinogenic risk assessments of oral exposure to nine nitrosamines were performed based on the monitoring results. EI-GC-MS/MS performed the best for the simultaneous analysis of these nine nitrosamines with respect to overall linearity, trace analysis limit of detection (less than 1 μg), recovery (average 108.66 ± 9.32%), and precision (less than 6%), compared with liquid chromatography-tandem mass spectrometry (LC-MS/MS) (APCI and ESI) methods. Using the EI-GC-MS/MS method, these nine nitrosamines eluted into artificial saliva from 54 rubber-type products were monitored. Based on the monitoring data, risk assessment was performed by calculating the margin of exposure (MOE) for the respective nitrosamines detected. As a result, these nitrosamines were confirmed to be safe with regard to both non-carcinogenic and carcinogenic risks.

## 1. Introduction

Nitrosamines are known to be produced by the reaction of amines occurring from the decomposition of additives, such as vulcanization accelerators used for the manufacture of rubber products and nitrites in the air or saliva [1,2]. These nitrosamines have been found to be carcinogenic through various animal experiments. The degree of carcinogenicity of nitrosamines varies depending on the chemical structure of the nitrosamines. In particular, n-nitrosodimethylamine (NDMA) and n-nitrosodiethylamine (NDEA), commonly detected in rubber manufacturing factories, were classified as 2A in 1987 by the International Agency for Research on Cancer (IARC) [3,4,5]. In addition, n-nitrosodi-n-propylamine (NDPA), n-nitrosodi-n-buthylamine (NDBA), n-nitrosopiperidine (NPIP), n-nitrosopyrrolidine (NPYR), and n-nitrosomorpholine (NMOR) are also designated as 2B [3].

Accordingly, in Korea, seven types of nitrosamines (NDMA, NDEA, NDPA, NDBA, NPIP, NPYR, NMOR) are set and managed to be 0.01 mg/kg or less as the dissolution standard from pacifiers according to the ‘Standards and Specifications for Instruments, Containers and Packaging’ [6]. In addition, artificial saliva should be used as the elution vehicle, and nitrosamines should be measured by liquid chromatography-tandem mass spectrometry (LC-MS/MS) according to the officially recognized test method for nitrosamines [6].

Internationally, the EC Commission Directive 93/11/ECC [7] in the European Union (EU) delineates the dissolution standards for nitrosamines eluted from the nipples of nursing bottles and soothers. In the EU, nitrosamines are regulated at 0.01 mg/kg in total, and artificial saliva prepared according to the guidelines of the annex of the Directive is used as an elution media and analyzed by gas chromatography (GC) [7]. Furthermore, in the U.S. and Canada, the dissolution standard for nitrosamines eluted from the nipples of baby bottles is set at 0.01 mg/kg as the total of nitrosamines by the FDA CPG Sec.500.450 [8] and SOR/2016-180 [9]. In both countries, nitrosamines are recommended to be measured by GC-MS [8,9]. Among Asian countries, except for Korea, the dissolution standards for nitrosamines are set for pacifiers in China according to GB 4806.2-2015 [10] and GB 28402-2012 [11]. A total of 11 types of nitrosamines are required to be detected through the standard test method, and the dissolution standard is regulated at 0.01 mg/kg as the sum of these 11 types of nitrosamines [10,11].

In most countries, nitrosamines eluted from rubber products are regulated according to the sum of several nitrosamines. Furthermore, most of the regulated nitrosamines overlap, and the threshold concentration is 0.01 mg/kg as the sum of nitrosamines in these countries. Interestingly, the test method for detecting nitrosamines differs across countries and is largely divided into GC methods and LC methods. However, which method is better has yet to be investigated.

In the past, the GC method mainly referred to the gas chromatography-thermal energy analysis (GC-TEA), which is a traditional method with a high selectivity for N-nitroso compounds using thermal decomposition [12]. However, since MS with a higher sensitivity than GC-TEA has been developed, GC-MS(MS) (single or tandem MS) has been applied to analyze nitrosamines in several studies, as well as in Method 521 of the U.S. EPA [13,14,15,16,17]. The LC method has been reported to enable the simultaneous measurement of nitrosamines eluted into artificial saliva and has been verified as an alternative to the traditional GC-TEA method of the EU [2]. Indeed, previous studies, as well as Korean guidelines, have successfully analyzed nitrosamines in various types of matrices (medicines, nipples, condoms, water, etc.) using LC-MS/MS [18,19,20,21,22,23,24].

When comparing the GC and LC methods for detecting nitrosamines reported in the previous studies, it was difficult to determine which method is more suitable for the simultaneous analysis of nitrosamines eluted from rubber products. In the case of pharmaceuticals and environmental samples, some studies have compared GC and LC methods simultaneously [19,25,26]. Yahaya et al. [27] provided a comprehensive review of the analytical methods for evaluating nitrosamines for various samples. However, no study has compared the analytical efficiency of the GC-MS/MS and LC-MS/MS methods for analyzing nitrosamines eluted into artificial saliva.

Here, we compared GC-MS/MS and LC-MS/MS for evaluating nine types of nitrosamines that are commonly regulated in many countries. The analytical methods were optimized by comparing the domestic and foreign nitrosamine analysis guidelines and the instrument conditions reported in previous studies. Then, the analytic efficiency was compared for linearity, limit of detection (LOD), limit of quantitation (LOQ), recovery rate, and precision using artificial saliva as an elution medium. In particular, the APCI and ESI sources were compared for the LC method. After selecting the best-performing analysis method, the nitrosamines eluted from rubber products and the TPE, TPU, and PU products with similar characteristics to rubber products that are sold in Korea were monitored. We compared our results with those of previous studies [19,22,28,29,30,31,32,33,34] to verify the feasibility of the selected method. Finally, based on the monitoring results, we conducted a health risk assessment for the oral exposure to the nine nitrosamines eluted from synthetic resins.

## 2. Materials and Methods

### 2.1. Materials and Reagents

N-nitrosodimethylamine (NDMA), n-nitrosodiethylamine (NDEA), n-nitrosodi-n-propylamine (NDPA), n-nitrosodi-n-buthylamine (NDBA), n-nitrosopiperidine (NPIP), n-nitrosopyrrolidine (NPYR), n-nitrosomorpholine (NMOR), n-nitrosodiphenylamine (NDPhA), n-nitroso-n-methylethylamine (NMEA), and n-nitrodi-n-propylamine-d14 (internal standard) were purchased from Sigma-Aldrich Korea (Cheoin-gu, Yongin, Korea). Methanol (HPLC grade), ethanol (HPLC grade), acetonitrile (HPLC grade), acetic acid, and n-heptane were purchased from Duksan Pure Chemicals Co. (Danwon-gu, Ansan, Korea). Ultra-pure water was prepared using an aquaMAXTM Ultra 370 series (YL Instruments, Anyang, Korea) water purification system (18.2 MΩ cm).

### 2.2. Instrumentation and Apparatus

#### 2.2.1. EI-GC-MS/MS

In this study, electron impact-GC-MS/MS (EI-GC-MS/MS) analyses were performed using Thermo EVO 8000 (Thermo Scientific, Waltham, MA, USA). An Agilent DB-WAX UI (30 m, 0.25 mm, 0.25 μm) column was used for the analyses, which were performed in splitless mode. The inlet temperature was set to 240 °C, the transfer line temperature to 240 °C, and the MS source temperature to 230 °C. The energy of the electron ionization (EI) for MS detection was set to 70 eV. The oven was initially held at 50 °C for 3 min, and the temperature was subsequently increased to 150 °C at 15 °C/min, 180°C at 10 °C/min, and 240 °C at 25 °C/min and held at 240 °C for 10 min. Single reaction monitoring (SRM) mode was used for screening analysis of nitrosamines. Xcalibur (Thermo Scientific, Waltham, MA, USA) and NIST/EPA/NIH mass spectral library Ver. 2.2 (NIST, Gaithersburg, MD, USA) software were used for data processing.

#### 2.2.2. LC-MS/MS

##### APCI-LC-MS/MS

A PerkinElmer Series 200 HPLC System (PerkinElmer, Waltham, MA, USA) equipped with an AB Sciex API4000 triple quadrupole mass spectrometer (AB Sciex, Framingham, MA, USA) was used to analyze nitrosamines. An Agilent Zorbax Eclipse Plus C18 column (3.0 × 150 mm, 3.5 µm) was installed in the instrument. The analysis was performed with 0.1% formic acid in distilled water (MP; A) and acetonitrile (MP; B) at a flow rate of 0.2 mL/min. The column temperature was set at 40 °C, and the injection volume was 10 μL. The mobile phase gradient was started at 20% B, a linear gradient was applied to increase the eluent B to 100% in 10 min, and the system was held at 100% B for 5 min (equilibrium time).

An atmospheric pressure chemical ionization (APCI) was used to ionize the nitrosamines. The ion source temperature was set at 330 °C. By applying the multiple reaction monitoring (MRM) mode, the precursor ions and product ions of the 9 nitrosamines were designated. The collision voltage was set at 17–27 V and the collision gas was nitrogen. The ionspray voltage was set to 5500 V, the nebulizer current (corona discharge) was set to 3 µA, the curtain gas pressure was set to 30, the ion source gas (1 and 2) was set to 30, the collision gas was set to 3, and the ion source gas pressure was set to 30. Analyst 1.6.2 (AB SCIEX, Mundelein, IL, USA) software was used for data processing.

##### ESI-LC-MS/MS

An Agilent 1290 ultra-high-performance liquid chromatography (UHPLC) (Agilent Technologies, Santa Clara, MA, USA) equipped with an Agilent 6490 triple quadrupole mass spectrometer (Agilent Technologies, Santa Clara, MA, USA) was used to analyze the nitrosamines. An Agilent Zorbax Eclipse Plus C18 column (3.0 × 150 mm, 3.5 µm) was installed in the instrument. The analysis was performed with 0.1% formic acid in distilled water (MP; A) and acetonitrile (MP; B) at a flow rate of 0.2 mL/min. The column temperature was set at 40 °C, and the injection volume was 10 μL. The mobile phase gradient was started at 20% B, a linear gradient was applied to increase eluent B to 100% in 10 min, and the system was held at 100% B for 5 min (equilibrium time). For these equipment conditions, the LC conditions were set identically for accurate comparison with APCI.

An electrospray ionization (ESI) was used to ionize the nitrosamines. The ion source temperature was set at 200 °C. By applying the multiple reaction monitoring (MRM) mode, the precursor ions and product ions of the 9 nitrosamines were designated. The collision voltage was set at 12–28 V, and the collision gas was nitrogen. The nebulizer pressure was set to 20 psi, the sheath gas temperature was set to 275 °C, and the gas flow rate was set to 11 L/min. The capillary voltage was set to 3500 V, and the sensitivity was increased by additionally setting the nozzle voltage to 1500 V. MassHunter Workstation Software (Agilent, Santa Clara, CA, USA) was used for data processing.

### 2.3. Samples

The number of samples to be monitored in this study was selected by classifying by material and considering the total ratio of domestic production and import. Samples of food contact materials were selected by reflecting the sales volume of large domestic markets and portal sites. For the selection of the target material, rubber material (*n* = 49) for which the standards for nitrosamines have been established in the “Standards and Specifications for Food Utensils, Containers and Packages” announced by the Korea Ministry of Food and Drug Safety [6] was preferentially selected. Among synthetic resin materials that control nitrosamines, materials that are difficult to elute and migrate because they do not come into direct contact with food were excluded from the target materials for analysis. Although there are no standards or specifications for nitrosamines in the standard test method, TPE (*n* = 2), TPU (*n* = 5), and PU (*n* = 1), which have similar appearances and characteristics to rubber, were added to the material to be analyzed. A total of 54 samples were purchased, and the purchased samples were dried and stored after removing only the external contaminants using running water for accurate experiments.

### 2.4. Preparation of the Standard Solutions

#### 2.4.1. Standard Solution

Twenty milligrams of n-nitrosodimethylamine (NDMA), n-nitrosodiethylamine (NDEA), n-nitrosodi-n-propylamine (NDPA), n-nitrosodi-n-buthylamine (NDBA), n-nitrosopiperidine (NPIP), n-nitrosopyrrolidine (NPYR), n-nitrosomorpholine (NMOR), n-nitrosodiphenylamine (NDPhA), and n-nitroso-n-methylethylamine (NMEA) were precisely weighed and dissolved in methanol to make 100 mL, which was used as the standard stock solution. The prepared standard solutions were refrigerated and protected from light. The prepared nitrosamine standard stock solution was diluted with artificial saliva and prepared in various concentration ranges, and finally the all method validation process was performed by applying the nitrosamine standard solution diluted with artificial saliva.

#### 2.4.2. Internal Standard Solution

Twenty milligrams of n-nitrodi-n-propylamine-d14 or n-nitrosodiisopropylamine was precisely weighed and dissolved in methanol to make 100 mL. One milliliter of this solution was placed in a 200 mL volumetric flask, and methanol was added to make 200 mL; this solution was used as an internal standard solution (the concentration of the internal standard solution was prepared at 1 µg/mL). All internal standard solutions were kept at 5 °C or lower after blocking light to prevent decomposition.

### 2.5. Preparation of the Sample

#### 2.5.1. Preparation of Artificial Saliva

Artificial saliva was manufactured using the method specified in the guidelines for the nitrosamine analysis of the European Commission and the Korean Ministry of Food and Drug Safety [6,7]. Nine hundred milliliters of distilled water was added to 4.2 g of sodium hydrogen carbonate, 0.5 g of sodium chloride, 0.2 g of potassium carbonate, and 30 mg of sodium nitrite, and the pH was adjusted to 9.0 using 0.1 N sodium hydroxide solution (or 0.1 N hydrochloric acid solution). Finally, distilled water was added to prepare 1 L of the solution as artificial saliva.

#### 2.5.2. Extraction of Nitrosamines

The nitrosamine test method of the “Standards and Specifications for Food Utensils, Containers and Packages” announced by the Korea Ministry of Food and Drug Safety [6] was referred to as a pretreatment method for nitrosamines. Among the nitrosamines, some of them, such as NDMA and NDEA, have sensitivity to light; thus, all the pretreatment processes used in this study were performed with light blocking. First, a synthetic resin sample was put in water, boiled for 10 min, cooled, and then cut into pieces. After the sample was dried, 10 g of the sample was weighed and then soaked in 40 mL of artificial saliva heated to 40 °C. The temperature was maintained and left for 24 h to elute nitrosamines into the artificial saliva. The eluate from which the nitrosamines were eluted was transferred to a 50 mL volumetric flask. Finally, the sample was washed with 5 mL of artificial saliva, added to the eluate, and adjusted to 50 mL with water.

#### 2.5.3. Pretreatment of Artificial Saliva

The artificial saliva in which the nitrosamines were eluted was pretreated according to the abovementioned Korean Food and Drug Administration guidelines [6]. Forty milliliters of artificial saliva, in which nitrosamine was eluted was accurately weighed and transferred to a separate funnel, and 0.5 mL of the internal standard solution and 1 mL of 0.1 N sodium hydroxide solution were added. Then, 20 mL of dichloromethane was added, and the solution was shaken for 5 min and then left still; the dichloromethane layer was then transferred to a Kudernadanish concentrator. Twenty milliliters of dichloromethane was added to the remaining solution, and the same procedure as previous was followed; the dichloromethane layer was combined with a Kudernadanish concentrator. Finally, 1 mL of methanol was added and mixed, nitrogen was slowly flowed at room temperature, and the solution concentrated to 1 mL was used as the sample solution.

### 2.6. Method Validation

The purpose of this study was to find the optimal method for analyzing nitrosamines in synthetic resins by verifying the analytical methods of EI-GC-MS/MS, APCI-LC-MS/MS, and ESI-LC-MS/MS for nitrosamines and to increase the reliability of monitoring results. The “Guidelines for performance criteria and validation procedures of analytical methods used in controls of food contact materials, 2009”, published by JRC, were referred to in order to verify the analytical method [35].

All method verification procedures were measured 3 times or more, and inter- and intra-day analyses were performed. In addition, for all verification procedures, standard solutions of nitrosamines were applied in various concentration ranges diluted with artificial saliva. To confirm the linearity of the analytical method, the standard solution (diluted with artificial saliva) was analyzed for each concentration to prepare a calibration curve, and the coefficient factor of the calibration curve was calculated. Regarding the LOD of the analytical method for nitrosamines, a standard solution for each concentration diluted in artificial saliva was pretreated according to the analytical method and analyzed, and the concentration of each substance with a signal-to-noise ratio of 3 or more was selected. In the case of the LOQ, the concentration was calculated at twice the LOD according to the method validation regulations of the JRC [35]. In this study, the recovery was measured by analyzing a sample prepared by adding 2 concentrations (low and high concentrations) of nitrosamine-mixed standard solution to artificial saliva. In addition, for reproducibility, inter-day and intra-day analyses were performed on samples prepared by independently adding 2 concentrations (low and high concentrations) of nitrosamine-mixed standard solution to artificial saliva, and the relative standard deviation (%RSD) was measured.

The verification results of the evaluated 3 analytical methods determined the suitability of the analysis by determining conformance or non-conformity based on the criteria proposed in the JRC guidelines [35].

### 2.7. Health Risk Assessment

#### 2.7.1. Exposure Assessment

The exposure assessment was performed by applying the “Guidance for Industry: Preparation of Premarket Submissions for Food Contact Substances (Chemistry Recommendations)” method published by the U.S. FDA [36]. Consumption factors and food-type distribution factors used in the exposure assessment were referenced to values appropriate to the situation in Korea [37]. The risk was assessed by calculating the estimated daily intake (EDI) by applying the concentrations of nitrosamines and comparing them with the benchmark dose lowest 10 (BMDL_10_). In this study, the total daily food intake and body weight were set to an average of 1.5 kg/person and 60 kg/person, respectively. The formula for calculating the EDI is as follows:

(1) $$Migration:<M>=\sum Concentration_{i}\;\left(mg/kg\right)\times Food\;type\;distribution\;factor\left(f_{T}\right)$$

(2) $$Dietary\;concentration\;of\;substances=Consumption\;factor\;\left(CF\right)\times <M>\left(mg/kg\right)$$

(3) $$EDI=\frac{Total\;daily\;food\;intake\;\left(kg/day\right)\times Dietary\;concentration\;of\;substance\;\left(mg/kg\right)}{Body\;weight\;\left(kg\;bw\right)}$$

#### 2.7.2. Non-Carcinogenic Risk Assessment: Margin of Exposure (MOE)

In this study, based on the calculated EDI of nitrosamines, the BMDL_10_ was investigated, and the MOE was calculated in the following manner to evaluate the non-carcinogenic risk. The MOE was calculated by dividing the irradiated BMDL_10_ by the estimated daily exposure level calculated from food intake (Equation (4)) [38]. The calculated MOE value confirmed the level of risk by applying the range of the MOE from the COC Annual Report [38].

(4) $$Margin\;of\;exposure\;\left(MOE\right)=\frac{Point\;of\;departure\;\left(POD\right)\;}{Estimated\;daily\;intake\;\left(EDI\right)}$$

#### 2.7.3. Carcinogenic Risk Assessment

In addition, in this study, the excess carcinogenic risk was evaluated for 9 nitrosamines, which is designated as a carcinogen. If the excess carcinogenic risk was lower than the safety tolerance level of 10^−6^, the initial risk assessment result indicated that the carcinogenic risk was negligible, and if it exceeded it, it was judged that it was not negligible. The excess carcinogenic risk was calculated by Equation (5).

(5) Carcinogenic risk=Daily exposure amount of the substances to be assessed×Cancer slope factor

## 3. Results and Discussion

### 3.1. Optimization of GC and LC Conditions for the Simultaneous Analysis of Nine Nitrosamines in Artificial Saliva

The analytical conditions for GC and LC were optimized by referring to previous studies on the simultaneous analysis of nitrosamines using GC-MS/MS and LC-MS/MS, and the nitrosamine analysis guidelines of domestic and overseas countries. In previous studies using GC [13,14,15,16,17], the WAX column in which the stationary phase is ethylene glycol is used most frequently in the analysis of nitrosamines. We selected the DB-WAX UI (30 m × 0.25 mm, 0.25 μm) column accordingly. The starting temperature was raised from 50 °C and passed through the midpoint of 150 °C and 180 °C, and the final temperature was set to 240 °C (the limit temperature of the column applied), referring to various conditions of previous studies on GC-MS(MS). Under these conditions, the nine nitrosamines were clearly separated, and the peak was very sharp. The tandem mass spectrometer used argon as the collision gas, and selected reaction monitoring (SRM) mode was applied. Noise was minimized, and product ions were confirmed according to the individual precursor ions of nine nitrosamines.

In the case of LC, the instrument conditions of previous studies that used the test method specified in Korean “Standards and Specifications for Utensils, Containers and Packages” [6] were reviewed. First, the separation was improved by optimizing the column and concentration gradient conditions, and finally, each mass spectrometer condition was optimized. In the case of the column, a 250 mm long column was used in the guideline, but we applied a 150 mm column for faster detection, which was confirmed to be suitable for the separation of the nine nitrosamines. The column temperature was set to 40 °C, and formic acid suitable for the positive mode was applied as solvent A of the mobile phase. In the case of solvent B, acetonitrile, which was the most widely used solvent in previous studies, was applied [18,19,20,21,22,23,24]. By comparing the concentration gradient conditions by testing various A:B ratios, the conditions of the standard test method of Korea were applied [6]. The optimized LC conditions were applied to both the APCI and ESI methods for comparison.

**Figure 1:**
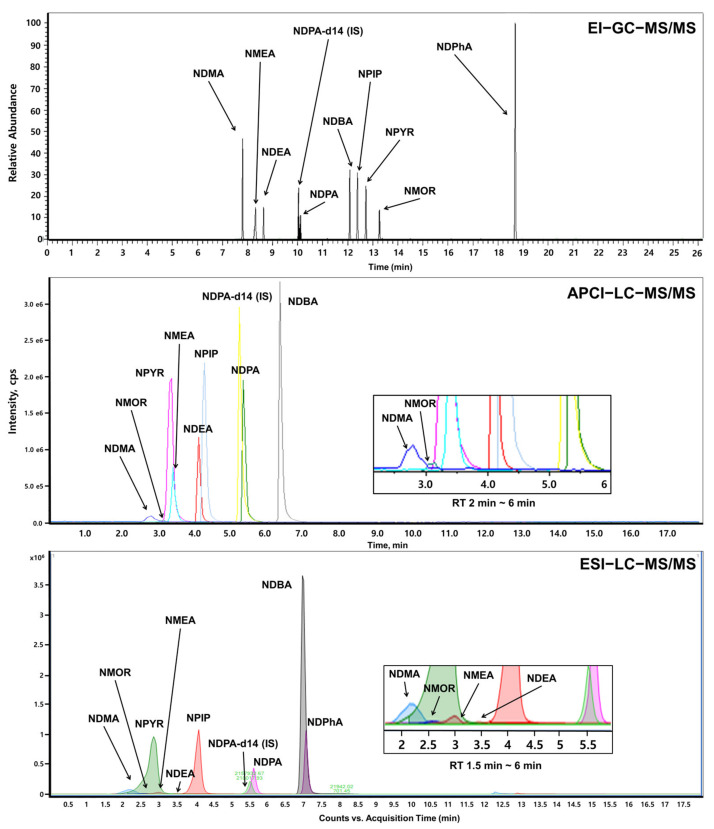
Chromatogram results for each analysis method of nine nitrosamine standard solutions prepared with artificial saliva.

In the case of mass spectrometers, both precursor ions and product ions were confirmed by applying the MRM mode, and the confirmed ion values were verified with the official test method of each country [6,23,24]. Each mass spectrometer maximized ionization by optimizing the source temperature, gas pressure of the nebulizer, and temperature and flow rate of dry gas. Finally, various capillary voltages were optimized to improve the sensitivity for the detection of the nine nitrosamines. The MRM for the nine nitrosamines was confirmed for the EI-GC-MS/MS, APCI-LC-MS/MS, and ESI-LC-MS/MS instrument conditions, as shown in Table 1, and the chromatograms of the nitrosamine standard solutions prepared with artificial saliva for each instrument condition are shown in Figure 1.

### 3.2. Method Validation of GC-MS/MS and LC-MS/MS for the Analysis of Nine Nitrosamines in Artificial Saliva

#### 3.2.1. Linearity

In this study, linearity was evaluated by applying a common concentration range to measure the correlation coefficients of the slopes of all analysis methods. For the concentration range, five or more points were selected according to the JRC guidelines [35]. The concentration range was set to 0.06–125 µg/L to comprehensively encompass trace to high concentrations (standard solution diluted with artificial saliva). The intra- (*n* = 3) and inter-day (*n* = 3) linearity was evaluated. In the case of intra-day, the analysis was performed three or more times within a day, and the inter-day analysis was performed once a day for 3 days.

In EI-GC-MS/MS, the correlation coefficient of calibration curves for nitrosamines (NDMA, NDEA, NDPA, NDBA, NPIP, NPYR, NMOR, NDPhA, NMEA) was measured to be 0.99 or higher. The correlation coefficient (*R^2^*) of all calibration curves for intra-day analysis (*n* = 3) and inter-day analysis (*n* = 3) was 0.99 or higher, suggesting an excellent linearity.

In the case of APCI-LC-MS/MS, as with EI-GC-MS/MS, the correlation coefficient of the inter-day and intra-day calibration curves of most nitrosamines was measured to be 0.99 or higher. However, NDPhA was not detected in APCI-LC-MS/MS and accordingly the linearity could not be measured. In the case of ESI-LC-MS/MS, the correlation coefficient of inter-day and intra-day calibration curves of all nitrosamines was measured to be 0.99 or higher. When comparing the three analysis methods, EI-GC-MS/MS and ESI-LC-MS/MS performed equally best in linearity, and reproducibility. The linearity results for the three analysis methods are shown in Table 2.

#### 3.2.2. LOD and LOQ

The LOD of EI was measured 0.12 µg/L for NDMA, 0.24 µg/L for NDEA, 0.24 µg/L for NDPA, 0.24 µg/L for NDBA, 0.12 µg/L for NPIP, 0.12 µg/L for NPYR, 0.24 µg/L for NMOR, 0.24 µg/L for NDPhA, and 0.48 µg/L for NMEA µg/L in artificial saliva. In the case of the LOQ, the standard of the JRC [35] in which the LOQ is calculated as twice of LOD, was applied. As a result, EI-GC-MS/MS was confirmed to have excellent sensitivity overall since all of the 9 nitrosamines had LOD and LOQ values of less than 1 µg/L, which were sufficient to analyze even trace amounts of nitrosamines.

The LOD of APCI-LC-MS/MS was measured to be 62.5 µg/L for NDMA, 0.48 µg/L for NDEA, 0.98 µg/L for NDPA, 0.49 µg/L for NDBA, 0.24 µg/L for NPIP, 0.48 µg/L for NPYR, 3.12 µg/L for NMOR, and 3.12 µg/L for NMEA in artificial saliva. The sensitivity for most of nitrosamines was relatively poor compared with EI-GC-MS/MS. NDPhA was not detected. In particular, NDMA, a human carcinogen, also showed a very poor LOD with APCI-LC-MS/MS compared with EI-GC-MS/MS. These results differed from the LOD of NDMA reported in a previous study using APCI [21]; this difference was presumed to be from the difference in the medium of artificial saliva.

The LOD of ESI-LC-MS/MS was measured to be 62.5 µg/L for NDMA, 7.81 µg/L for NDEA, 7.81 µg/L for NDPA, 0.24 µg/L for NDBA, 0.48 µg/L for NPIP, 0.24 µg/L for NPYR, 0.24 µg/L for NMOR, 0.24 µg/L for NDPhA, and 62.5 µg/L for NMEA in artificial saliva. ESI-LC-MS/MS detected all 9 nitrosamines including NDPhA, reflecting that ESI is a more suitable method for the simultaneous analysis of nitrosamines than APCI. However, the overall sensitivity was poorer than that of EI-GC-MS/MS. In conclusion, EI-GC-MS/MS was the most sensitive and reliable method for the simultaneous analysis of 9 nitrosamines. The LOD and LOQ values for the three methods are shown in Table 3.

#### 3.2.3. Recovery (Accuracy)

The recovery rate of the nitrosamines in EI-GC-MS/MS was measured at spike concentrations of 1 µg/L and 5 µg/L. The recovery rate of NDMA, NDEA, NDPA, NDBA, NPIP, NPYR, NMOR, NDPhA, and NMEA was 87.26% to 109.64%, 103.43% to 121.55%, 101.36% to 110.42%, 107.91% to 120.03%, 103.34% to 115.70%, 97.50% to 111.78%, 94.55% to 108.06%, 117.83% to 133.52%, and 99.72% to 109.16%, respectively. It was confirmed that the recoveries of all nitrosamines except for NDPhA met the JRC standards (for an addition of samples of less than 10 µg/L, an average recovery rate of 40% to 120%, should be obtained [35]).

The recovery rate for APCI-LC-MS/MS and ESI-LC-MS/MS was evaluated at spike concentrations of 10 µg/L and 100 µg/L, considering the poorer LOD than EI-GC-MS/MS. First, in the case of APCI-LC-MS/MS, the recovery rate for NDMA, NDEA, NDPA, NDBA, NPIP, NPYR, and NMOR was 83.45% to 109.34%, 97.43% to 121.55%, 95.73% to 110.42%, 99.62% to 120.03%, 94.97% to 115.70%, 97.94% to 109.69%, and 86.28% to 105.96%, respectively. NDPhA was not detected, and the recovery of NMEA was 96.06% to 140.83%, confirming that most of the nitrosamines, except for the undetected NDPhA, satisfied the criteria of the JRC standard.

In the case of ESI-LC-MS/MS, the recovery rate of NDMA, NDEA, NDPA, NDBA, NPIP, NPYR, NMOR, NDPhA, and NMEA was 67.82% to 93.54%, 71.99% to 85.55%, 81.96% to 117.77%, 93.14% to 110.06%, 76.47% to 118.55%, 84.81% to 111.02%, 74.66% to 123.59%, 76.08% to 123.59%, and 75.35% to 94.57%, respectively, confirming that the average recovery rates of all 9 nitrosamines met the JRC standard. When comparing the lowest recovery of the three analytical methods, that of EI-GC-MS/MS was 101.41 ± 8.75%, that of APCI-LC-MS/MS was 93.94 ± 5.83% (excluding NDPhA), and that of ESI-LC-MS/MS was 79.81 ± 9.42%, suggesting that EI-GC-MS/MS showed better recovery than LC-MS/MS methods even at lower spiking concentrations. The detailed results at two concentrations (low and high) for the three analytical methods are shown in Table 4.

Overall, when the recovery results for the three analytical methods were compared, the LC-MS/MS methods had poor LOD values, NDPhA was not detected (APCI), or there was a large deviation in recovery (ESI). On the other hand, EI-GC-MS/MS showed a stable recovery rate even at a small spike concentration of 1 µg/L and showed a good reproducibility of less than approximately 10% in the case of repeatability. Therefore, GC-MS/MS was judged to be more suitable for the simultaneous analysis of the nine types of nitrosamines than LC-MS/MS.

#### 3.2.4. Precision

The precision was measured by calculating the RSD of the recovery measurement results obtained with the three analytical methods. As a result, EI-GC-MS/MS exhibited a minimum precision of 0.82% to a maximum of 3.90% RSD at a low concentration of 1 µg/L and a minimum precision of 1.25% to a maximum of 6.26% at a high concentration of 5 µg/L. Overall, the precision was confirmed to be less than 6%, meeting the precision verification standard of the JRC (%RSD < 22.6% for a sample < 100 µg/L) [35].

APCI-LC-MS/MS showed a minimum precision of 1.96% to a maximum of 13.44% RSD at a low concentration of 10 µg/L, and at a high concentration of 100 µg/L, this method showed a minimum precision of 0.92% to a maximum of 7.63% RSD (excluding NDPhA). In the case of ESI-LC-MS/MS, at a low concentration of 10 µg/L, the precision was a minimum of 4.60% to a maximum of 11.80% RSD (excluding NDMA and NDEA due to the poor LOD), and at a high concentration of 100 µg/L, the precision was a minimum of 2.28% to a maximum of 12.55%, confirming that these LC-MS/MS results also met both the JCR standard criteria of 22.6% (less than 100 ppb) and 16.0% (less than 1 ppm) [35]. However, the LC method generally showed a large variation between the minimum and maximum RSDs, and some substances exhibited borderline values even though the spike concentration was higher than that with EI-GC-MS/MS, suggesting that the EI-GC-MS/MS analysis method is more suitable for the simultaneous analysis of the nine nitrosamines. The detailed precision measurement results for the three analytical methods are shown in Table 5.

### 3.3. Measurement of Nitrosamines Eluted from Synthetic Resin into Artificial Saliva with EI-GC-MS/MS

#### 3.3.1. Classification by Product Type

The elution of nitrosamines into artificial saliva was measured for rubber, TPE, TPU, and polyurethane products using an EI-GC-MS/MS method. Forty-nine rubber samples and eight samples made of TPE, TPU, and PU samples were pots, storage containers, tableware, cooking utensils, cups, cutting boards, and nipples of baby bottles. Of the 49 rubber samples, nitrosamines were detected in 36 products up to 3.40 µg/L as total nitrosamines, confirming that they were acceptable for Korean standards and specifications (<10 µg/kg). In case of TPE, TPU, and PU samples, trace amounts of nitrosamines were detected in 1 out of 1 PU sample up to a maximum concentration of 0.92 µg/kg, confirming that they were acceptable for Korean standards and specifications.

#### 3.3.2. Classification by Nitrosamines

**Figure 2:**
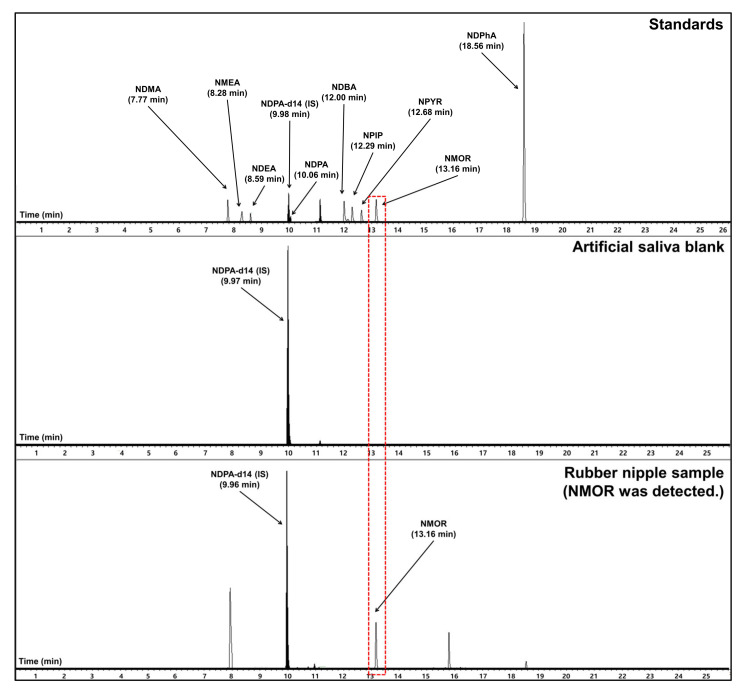
Representative chromatogram of a rubber nipple sample with nitrosamine (NMOR) detected.

In artificial saliva, NDMA, NDEA, NDPA, NDBA, NPIP, NPYR, NMOR, NDPhA, and NMEA were detected in 2 out of 57 products, 3 out of 57 products, 0 out of 57 products, 10 out of 57 products, 12 out of 57 products, 2 out of 57 products, 31 out of 57 products, 9 out of 57 products, and 0 out of 57 products, respectively. NMOR was detected in almost all products. Among samples in which NMOR was detected, a representative chromatogram for the rubber nipple product is shown in Figure 2. Of note, NDMA, which is a potential human carcinogen, was detected in two products.

#### 3.3.3. Comparison of the Results with Those of Previous Studies

Our monitoring data were compared with the previous studies (Table 6). A total of nine previous studies were compared [19,22,28,29,30,31,32,33,34]. The concentration ranges and average concentrations of nitrosamines reported in previous studies were compared, and if the average detection concentrations were not given, the average of the maximum and minimum detection values was used for comparison. In the case of TPE, TPU, and PU samples, there were no studies reporting nitrosamine elution.

In the case of the rubber samples, trace amounts of NDMA, NDBA, NPIP, NMOR, NDPhA, and NMEA were detected in previous studies [19,22,28,29,30,31,32,33,34], and similar levels were detected in our study. For NDEA, the monitoring results of this study were relatively higher than those of previous studies. NDPA was not detected in our study or in previous studies. NPYR was detected in trace amounts by Anna, V. et al. [28]. The monitoring result of this study was relatively higher but fell within a similar range. Collectively, we can confirm that our EI-GC-MS/MS method is well-suited for the analysis of nitrosamine elution from synthetic resins. NMOR was found to be detected in trace amounts in previous studies and was confirmed to be at a level similar to the monitoring results of this study. NDPhA was found to be detected in trace amounts in the study by Kühne, F. et al. [31] and was confirmed to be at a level similar to the monitoring results of this study. NMEA was found to be detected in trace amounts in previous studies and was confirmed to be at a level similar to the monitoring results of this study. We also performed comparisons with other types of samples. The results of a previous study monitoring nitrosamines in elastomer raw material for rubber production were slightly higher than the results of monitoring nitrosamines in the food contact material of this study.

### 3.4. Risk Assessment

#### 3.4.1. Non-Carcinogenic Risk Assessment

We conducted a non-carcinogenic risk assessment for the oral exposure to nitrosamines based on BMDL_10_. Among the nine nitrosamines we monitored the BMDL_10_ for NDMA, NDEA, NPYR, and NMOR. The daily intake (EDI) was estimated by considering our monitoring results of nitrosamines, the MOE was calculated based on the reported BMDL_10_, and the risk was evaluated. The calculated MOE was applied to the scope of the exposure safety margin through the COC annual report [38] to confirm the risk level. We also evaluated the potential risk by considering the average LOD value for each nitrosamine for the samples in which nitrosamines were not detected.

All the assessed nitrosamines (NDMA, NDEA, NPYR, and NMOR) were confirmed to have MOEs of 100,000 or more, indicating no potential harmful effects. However, the risk of rubber material was approximately 10 times higher than that of other materials. Therefore, continuous monitoring and management of rubber products is necessary. Table 7 shows the non-carcinogenic risk assessment results for each nitrosamine.

#### 3.4.2. Carcinogenic Risk Assessment

The cancer slope factor for the in nitrosamines was investigated to evaluate their carcinogenic risks. By referring to the “Integrated Risk Information System (IRIS) Chemical Assessment Summary” of the U.S. EPA and California Office of Environmental Health Hazard Assessment (OEHHA) data, the oral slope factor of NDMA, NDEA, NDPA, NDBA, NPIP, NPYR, NMOR, NDPhA, and NMEA was confirmed to be 5.1 × 10^+1^ mg/kg/day, 1.50 × 10^+02^ mg/kg/day, 7.00 × 10^+00^ mg/kg/day, 5.40 × 10^+00^ mg/kg/day, 9.40 × 10^+00^ mg/kg/day, 2.10 × 10^+00^ mg/kg/day, 6.70 × 10^+00^ mg/kg/day, 4.90 × 10^−03^ mg/kg/day, and 2.20 × 10^+01^ mg/kg/day, respectively. The carcinogenic risk was calculated by multiplying the EDI values of the nine nitrosamines calculated for each material by the oral slope factor. As a result of evaluating the carcinogenic risk of these nine nitrosamines in each material, it was confirmed that there was no carcinogenic risk because all materials showed values of 10^−4^–10^−6^ or less.

However, overall, the carcinogenic risk of rubber and TPE material was measured to be relatively high compared with the risk of other materials. NDEA showed a relatively high carcinogenic risk compared with other nitrosamines at a level of 10^−5^ in rubber and TPE, reflecting that the carcinogenic risk of NDEA in rubber and TPE might not be negligible. However, since the calculated risk was relatively high by adding the potential risk obtained by applying the LOD value to the sample in which nitrosamine was not detected, there was practically no carcinogenic risk. Table 8 shows the carcinogenic risk assessment results for each nitrosamine.

## 4. Conclusions

In this study, we compared the analytic efficiency of EI-GC-MS/MS, APCI-LC-MS/MS, and ESI-LC-MS/MS for analyzing nitrosamines eluted from food contact materials into artificial saliva. We demonstrated that EI-GC-MS/MS showed superior results in the validation test compared with LC-MS/MS methods. In the case of EI-GC/MS, the overall sensitivity of nitrosamines was superior to that of LC-MS/MS, and the ionization efficiency was relatively high (especially in the case of NDMA). LC-MS/MS did not show a significant difference in ionization efficiency overall between APCI and ESI sources, but the fact that NDPhA was not analyzed in the APCI source was judged to be disadvantageous in the simultaneous analysis, which requires additional research. In addition, we conducted the monitoring of nitrosamines eluted from TPE, TPU, and PU samples with rubber-like properties, including rubber, by applying the selected EI-GC-MS/MS method, which produced similar results to those of previous studies, demonstrating that the EI-GC-MS/MS method is suitable for monitoring nitrosamine elution from synthetic resins. Risk assessment was performed based on the nitrosamine monitoring results, which demonstrated that synthetic resin products currently distributed in the Korea market were safe because they showed little or no non-carcinogenic and carcinogenic risk. Our study results could be useful for other fields for the efficient management of nitrosamines.

## Figures and Tables

**Table 1:** SRM and MRM results of nine nitrosamines established by each analysis method (retention time, precursor ion, product ion, and CE).

Nitrosamines	R.T (min), Average ± SD (%RSD)		Precursor Ion	Product Ion	CE (V)
	Intra-Day (*n* = 3)	Inter-Day (*n* = 3)			
**EI-GC-MS/MS**					
NDMA	7.78 ± 0.03 (0.34)	7.76 ± 0.03 (0.32)	74.1	44.0	5
NDEA	8.60 ± 0.03 (0.37)	8.57 ± 0.04 (0.51)	102.1	44.1	10
NDPA	10.10 ± 0.04 (0.37)	10.05 ± 0.03 (0.32)	130.0	43.0-	10
NDBA	12.06 ± 0.05 (0.41)	11.99 ± 0.03 (0.22)	116.1	99.1	15
NPIP	12.34 ± 0.05 (0.41)	12.29 ± 0.03 (0.26)	114.0	84.1	5
NPYR	12.68 ± 0.04 (0.32)	12.62 ± 0.03 (0.23)	100.1	55.1	5
NMOR	13.21 ± 0.06 (0.42)	13.16 ± 0.03 (0.24)	86.1	56.1	15
NDPhA	18.61 ± 0.07 (0.39)	18.54 ± 0.03 (0.14)	169.1	168.1	10
NMEA	8.29 ± 0.02 (0.28)	8.27 ± 0.02 (0.24)	88.1	42.1	10
NDPA-d_14_	10.03 ± 0.04 (0.36)	9.99 ± 0.01 (0.06)	78.1	46.1	15
**APCI-LC-MS/MS**					
NDMA	2.75 ± 0.04 (1.28)	2.81 ± 0.07 (2.57)	75.2	43.0	23
NDEA	4.12 ± 0.01 (0.14)	4.12 ± 0.01 (0.14)	103.2	75.0	17
NDPA	5.35 ± 0.00 (0.00)	5.35 ± 0.01 (0.19)	131.2	89.0	15
NDBA	6.37 ± 0.01 (0.18)	6.36 ± 0.01 (0.09)	159.3	57.0	23
NPIP	4.26 ± 0.01 (0.14)	4.27 ± 0.01 (0.27)	115.2	64.2	23
NPYR	3.34 ± 0.01 (0.17)	3.34 ± 0.01 (0.17)	101.2	55.0	25
NMOR	3.10 ± 0.04 (1.22)	3.10 ± 0.03 (1.04)	117.2	87.0	19
NDPhA	-	-	199.2	169.2	25
NMEA	3.41 ± 0.03 (0.74)	3.43 ± 0.05 (1.32)	89.2	61.0	17
NDPA-d_14_	5.25 ± 0.01 (0.11)	5.24 ± 0.01 (0.11)	145.1	50.1	17
**ESI-LC-MS/MS**					
NDMA	2.15 ± 0.04 (2.07)	2.15 ± 0.04 (1.68)	75.1	43.0	18
NDEA	3.80 ± 0.03 (0.70)	3.81 ± 0.03 (0.66)	103.2	75.0	8
NDPA	5.61 ± 0.01 (0.20)	5.56 ± 0.05 (0.91)	131.2	43.0	12
NDBA	7.06 ± 0.05 (0.75)	7.02 ± 0.01 (0.14)	159.3	56.9	12
NPIP	4.07 ± 0.06 (1.35)	4.07 ± 0.03 (0.85)	115.2	41.0	22
NPYR	2.78 ± 0.03 (0.91)	2.80 ± 0.03 (1.09)	101.1	54.9	12
NMOR	2.54 ± 0.04 (1.42)	2.52 ± 0.03 (1.05)	117.1	87.0	8
NDPhA	7.05 ± 0.04 (0.59)	7.11 ± 0.01 (0.08)	199.2	65.9	15
NMEA	3.05 ± 0.05 (1.64)	3.07 ± 0.04 (1.36)	89.2	61.0	8
NDPA-d_14_	5.52 ± 0.03 (0.48)	5.50 ± 0.02 (0.36)	145.1	50.1	8

**Table 2:** Intra- and inter-day linearity of the three nitrosamine analysis methods.

NitrosamInes	EI-GC-MS/MS						APCI-LC-MS/MS						ESI-LC-MS/MS					
	Correlation Coefficients of the Slopes (*R^2^*)																	
	Intra-Day (*n* = 3)			Inter-Day (*n* = 3)			Intra-Day (*n* = 3)			Inter-Day (*n* = 3)			Intra-Day (*n* = 3)			Inter-Day (*n* = 3)		
	1	2	3	1	2	3	1	2	3	1	2	3	1	2	3	1	2	3
**NDMA**	0.9998	1.0000	0.9999	0.9999	0.9998	0.9995	0.9988	0.9975	0.9991	0.9992	0.9990	0.9995	0.9965	0.9988	0.9979	0.9982	0.9970	0.9992
**NDEA**	0.9997	0.9998	0.9998	0.9998	0.9999	0.9996	0.9999	0.9995	0.9997	0.9999	1.0000	0.9994	0.9984	0.9952	0.9973	0.9968	0.9970	0.9980
**NDPA**	0.9994	0.9998	0.9996	0.9996	0.9995	0.9995	0.9995	0.9992	0.9989	0.9987	0.9981	0.9999	1.0000	0.9999	0.9998	0.9997	1.0000	0.9996
**NDBA**	0.9997	0.9995	0.9997	0.9997	0.9996	0.9999	0.9991	0.9999	0.9995	0.9998	0.9992	0.9995	0.9999	0.9999	0.9998	0.9991	0.9990	0.9999
**NPIP**	0.9999	0.9994	0.9998	0.9995	0.9999	0.9998	0.9998	0.9979	0.9997	0.9982	0.9979	0.9988	0.9998	0.9997	0.9995	0.9999	1.0000	0.9992
**NPYR**	0.9994	0.9997	0.9995	0.9994	0.9997	0.9999	0.9986	0.9999	0.9992	0.9991	0.9993	0.9999	0.9995	0.9992	0.9999	0.9999	0.9991	0.9992
**NMOR**	0.9995	0.9999	0.9996	0.9999	0.9994	0.9999	1.0000	0.9999	0.9998	0.9992	0.9993	0.9980	0.9992	0.9991	0.9990	0.9998	0.9995	0.9989
**NDPhA**	0.9967	0.9999	0.9998	0.9987	0.9996	0.9999	-	-	-	-	-	-	0.9988	0.9999	0.9993	0.9995	1.0000	0.9991
**NMEA**	0.9988	0.9999	0.9999	0.9997	0.9998	0.9997	0.9968	0.9992	0.9978	0.9991	0.9989	0.9982	0.9999	0.9999	0.9999	0.9999	0.9998	0.9998

**Table 3:** Limits of detection (LOD) and limit of quantification (LOQ) of three nitrosamine analytical methods.

Nitrosamines	LOD (µg/L)			LOQ (µg/L)		
	GC-MS/MS	APCI-LC-MS/MS	ESI-LC-MS/MS	GC-MS/MS	APCI-LC-MS/MS	ESI-LC-MS/MS
**NDMA**	0.12	62.5	62.5	0.24	125	125
**NDEA**	0.24	0.48	7.81	0.48	0.96	15.62
**NDPA**	0.24	0.98	7.81	0.48	1.96	15.62
**NDBA**	0.12	0.49	0.24	0.24	0.98	0.48
**NPIP**	0.12	0.24	0.48	0.24	0.48	0.96
**NPYR**	0.12	0.48	0.24	0.24	0.96	0.48
**NMOR**	0.24	3.12	0.24	0.48	6.25	0.48
**NDPhA**	0.24	N.D.	0.24	0.24	-	0.48
**NMEA**	0.48	3.12	62.5	0.98	6.25	125

**Table 4:** Detailed recovery results of three nitrosamine analytical methods (low and high concentrations).

Nitrosamines	EI-GC-MS/MS		APCI-LC-MS/MS		ESI-LC-MS/MS	
	Spike Conc. (µg/L)	Avr. Recovery (%)	Spike Conc. (µg/L)	Avr. Recovery (%)	Spike Conc. (µg/L)	Avr. Recovery (%)
**NDMA**	**1**	105.46	**10**	-^1)^	**10**	-
	**5**	88.53	**100**	90.12	**100**	79.26
**NDEA**	**1**	115.33	**10**	110.83	**10**	73.50
	**5**	105.82	**100**	110.98	**100**	75.36
**NDPA**	**1**	109.40	**10**	108.93	**10**	90.52
	**5**	102.86	**100**	101.54	**100**	114.75
**NDBA**	**1**	117.39	**10**	111.45	**10**	96.47
	**5**	111.46	**100**	115.76	**100**	100.35
**NPIP**	**1**	113.20	**10**	106.76	**10**	83.60
	**5**	106.33	**100**	106.80	**100**	108.49
**NPYR**	**1**	110.07	**10**	103.87	**10**	93.51
	**5**	99.97	**100**	97.42	**100**	102.16
**NMOR**	**1**	105.11	**10**	101.16	**10**	87.42
	**5**	99.01	**100**	93.77	**100**	114.31
**NDPhA**	**1**	127.57	**10**	N.D.	**10**	80.60
	**5**	126.23	**100**	N.D.	**100**	123.47
**NMEA**	**1**	107.16	**10**	106.55	**10**	83.66
	**5**	104.98	**100**	131.76	**100**	83.28

^1)^ Not detected at that spike concentration.

**Table 5:** Detailed precision results of three nitrosamine analytical methods (low and high concentrations).

Nitrosamines	EI-GC-MS/MS		APCI-LC-MS/MS		ESI-LC-MS/MS	
	Spike Conc. (µg/L)	%RSD (%)	Spike Conc. (µg/L)	%RSD (%)	Spike Conc. (µg/L)	%RSD (%)
**NDMA**	**1**	3.90	**10**	-^1)^	**10**	-
	**5**	1.25	**100**	5.20	**100**	12.55
**NDEA**	**1**	2.34	**10**	11.08	**10**	10.42
	**5**	1.96	**100**	0.92	**100**	11.94
**NDPA**	**1**	0.82	**10**	1.96	**10**	10.28
	**5**	1.78	**100**	4.97	**100**	2.28
**NDBA**	**1**	0.88	**10**	9.50	**10**	4.60
	**5**	5.49	**100**	2.54	**100**	8.70
**NPIP**	**1**	2.10	**10**	9.98	**10**	8.60
	**5**	4.05	**100**	2.07	**100**	8.38
**NPYR**	**1**	1.64	**10**	5.66	**10**	12.84
	**5**	4.49	**100**	7.48	**100**	8.42
**NMOR**	**1**	3.48	**10**	4.63	**10**	13.19
	**5**	4.12	**100**	6.98	**100**	7.48
**NDPhA**	**1**	3.07	**10**	N.D.	**10**	9.70
	**5**	6.26	**100**	N.D.	**100**	7.51
**NMEA**	**1**	3.03	**10**	9.38	**10**	11.80
	**5**	4.40	**100**	7.63	**100**	6.51

^1)^ Not detected at that spike concentration.

**Table 6:** Comparison of the monitoring results of our study with those of previous studies.

Resins	Nitrosamines	Previous Studies	Analytical Method	Sample	Detection Frequency	Monitoring Results in Previous Studies (µg/kg)	Monitoring Results in our Study (µg/kg) (Detection Frequency)
**Rubber** **(*n* = 49)**	**NDMA**	KMFDS., 2009	LC-MS/MS	Nipples	0/349	ND	1.01~1.71 (2/49)
		Bouma, K., et al., 2003	GC-TEA	Nipples	15/19	0.20~1.60	
		Anna, V., et al., 2011	GC-MS/MS	Nipples	2/2	0.30~1.90	
		Mutsuga, M., et al., 2013	GC-MS	Nipples	0/3	ND	
		Suh et al., 2017	PCI-GC-MS/MS	Nipples	0/93	ND	
		Kühne, F., et al., 2018	APCI-LC-MS/MS	Elastomer	18/96	0.72~5.22	
		Park, S.J., et al., 2018	LC-MS/MS	Nipples and baby products, kitchenware	17/75	1.02~3.67	
	**NDEA**	KMFDS., 2009	LC-MS/MS	Nipples	0/349	ND	0.33~0.56 (3/49)
		Anna, V., et al., 2011	GC-MS/MS	Nipples	0/2	ND	
		Mutsuga, M., et al., 2013	GC-MS	Nipples	0/3	ND	
		Suh et al., 2017	PCI-GC-MS/MS	Food packaging	0/93	ND	
		Park, S.J., et al., 2018	LC-MS/MS	Nipples and baby products, kitchenware	0/75	ND	
	**NDPA**	KMFDS., 2009	LC-MS/MS	Nipples	0/349	ND	ND (0/49)
		Anna, V., et al., 2011	GC-MS/MS	Nipples	0/2	ND	
		Mutsuga, M., et al., 2013	GC-MS	Nipples	0/3	ND	
		Park, S.J. et al., 2018	LC-MS/MS	Nipples and baby products, kitchenware	0/75	ND	
	**NDBA**	KMFDS., 2009	LC-MS/MS	Nipples	0/349	ND	0.12~0.64 (10/49)
		Anna, V., et al., 2011	GC-MS/MS	Nipples	0/2	ND	
		Mutsuga, M., et al., 2013	GC-MS	Nipples	0/3	ND	
		Kühne, F., et al., 2018	APCI-LC-MS/MS	Rubber elastomer	18/96	0.54~2.04	
		Park, S.J., et al., 2018	LC-MS/MS	Nipples and baby products, kitchenware	0/75	ND	
		Suh et al., 2017	PCI-GC-MS/MS	Food packaging	0/93	ND	
	**NPIP**	KMFDS., 2009	LC-MS/MS	Nipples	0/349	ND	0.13~0.67 (12/49)
		Anna, V., et al., 2011	GC-MS/MS	Nipples	0/2	ND	
		Mutsuga. M., et al., 2013	GC-MS	Nipples	0/3	ND	
		Park, S.J., et al., 2018	LC-MS/MS	Nipples and baby products, kitchenware	3/75	0.38~0.55	
	**NPYR**	KMFDS., 2009	LC-MS/MS	Nipples	0/349	ND	0.12~0.15 (2/49)
		Anna, V., et al., 2011	GC-MS/MS	Nipples	1/2	0.6	
		Mutsuga, M., et al., 2013	GC-MS	Nipples	0/3	ND	
		Park, S.J., et al., 2018	LC-MS/MS	Nipples and baby products, kitchenware	0/75	ND	
	**NMOR**	KMFDS., 2009	LC-MS/MS	Nipples	0/349	ND	0.29~2.77 (29/49)
		Anna, V., et al., 2011	GC-MS/MS	Nipples	1/2	0.2	
		Mutsuga, M., et al., 2013	GC-MS	Nipples	0/3	ND	
		Kühne, F., et al., 2018	APCI-LC-MS/MS	Rubber elastomer	18/96	0.30~1.50	
		Park, S.J., et al., 2018	LC-MS/MS	Nipples and baby products, kitchenware	4/75	0.89~1.96	
	**NDPhA**	Anna, V., et al., 2011	GC-MS/MS	Nipples	2/2	0.1	0.27~1.88 (9/49)
		Kühne, F., et al., 2018	APCI-LC-MS/MS	Rubber elastomer	3/96	0.42~1.50	
		Zhao, Y.Y., et al., 2006	ESI-LC-MS/MS	River water	3/4	0.0006~0.0010	
	**NMEA**	Anna, V., et al., 2011	GC-MS/MS	Nipples	0/2	ND	ND (0/49)
		Zhao, Y.Y., et al., 2006	ESI-LC-MS/MS	River water	0/4	ND	
		Wang, X., et al., 2016	ESI-LC-MS/MS	River water	1/17	1.00	
**TPE, TPU, PU** **(*n* = 8)**	**NDMA**	-^1)^	-	-	-	-	ND (0/8)
	**NDEA**						ND (0/8)
	**NDPA**						ND (0/8)
	**NDBA**						ND (0/8)
	**NPIP**						ND (0/8)
	**NPYR**						ND (0/8)
	**NMOR**						0.92 (1/8)
	**NDPhA**						ND (0/8)
	**NMEA**						ND (0/8)

^1)^ There is no previous study data.

**Table 7:** Non-carcinogenic risk assessment results for four nitrosamines using the calculated margin of exposure (MOE).

Nitrosamines	Synthetic Resin	EDI (mg/kg bw/day)	BMDL_10_ (mg/kg bw/day)	MOE ^1)^
**NDMA**	Rubber	8.10 × 10^−08^	0.027	333,165
	TPE	5.70 × 10^−08^		473,684
	TPU	6.00 × 10^−09^		4,500,000
	PU	6.00 × 10^−09^		4,500,000
**NDEA**	Rubber	1.19 × 10^−07^	0.018	150,963
	TPE	1.14 × 10^−07^		157,895
	TPU	1.20 × 10^−08^		1,500,000
	PU	1.20 × 10^−08^		1,500,000
**NPYR**	Rubber	5.73 × 10^−08^	0.16	2,792,769
	TPE	5.70 × 10^−08^		2,807,018
	TPU	6.00 × 10^−09^		26,666,667
	PU	6.00 × 10^−09^		26,666,667
**NMOR**	Rubber	2.99 × 10^−07^	0.7	2,342,976
	TPE	1.14 × 10^−07^		6,140,351
	TPU	1.20 × 10^−08^		58,333,333
	PU	4.60 × 10^−08^		15,217,391

^1)^ 10,000 or less: there is a possibility that harmful effects may occur; 10,000–100,000: the harmful effect is low; 100,000–1,000,000: there is little harmful effect; 1,000,000 or more: no harmful effects.

**Table 8:** Carcinogenic risk assessment results for nine nitrosamines.

Nitrosamines	Synthetic Resin	EDI (mg/kg bw/day)	Cancer Slope Factor (mg/kg bw/day)	Carcinogenic Risk
**NDMA**	Rubber	8.10 × 10^−08^	5.10 × 10^+01^	4.13 × 10^−06^
	TPE	5.70 × 10^−08^		2.91 × 10^−06^
	TPU	6.00 × 10^−09^		3.06 × 10^−07^
	PU	6.00 × 10^−09^		3.06 × 10^−07^
**NDEA**	Rubber	1.19 × 10^−07^	1.50 × 10^+02^	1.79 × 10^−05^
	TPE	1.14 × 10^−07^		1.71 × 10^−05^
	TPU	1.20 × 10^−08^		1.80 × 10^−06^
	PU	1.20 × 10^−08^		1.80 × 10^−06^
**NDPA**	Rubber	1.14 × 10^−07^	7.00 × 10^+00^	7.98 × 10^−07^
	TPE	1.14 × 10^−07^		7.98 × 10^−07^
	TPU	1.20 × 10^−08^		8.40 × 10^−08^
	PU	1.20 × 10^−08^		8.40 × 10^−08^
**NDBA**	Rubber	8.32 × 10^−08^	5.40 × 10^+00^	4.49 × 10^−07^
	TPE	5.70 × 10^−08^		3.08 × 10^−07^
	TPU	6.00 × 10^−09^		3.24 × 10^−08^
	PU	6.00 × 10^−09^		3.24 × 10^−08^
**NPIP**	Rubber	7.29 × 10^−08^	9.40 × 10^+00^	6.85 × 10^−07^
	TPE	5.70 × 10^−08^		5.36 × 10^−07^
	TPU	6.00 × 10^−09^		5.64 × 10^−08^
	PU	6.00 × 10^−09^		5.64 × 10^−08^
**NPYR**	Rubber	5.73 × 10^−08^	2.10 × 10^+00^	1.20 × 10^−07^
	TPE	5.70 × 10^−08^		1.20 × 10^−07^
	TPU	6.00 × 10^−09^		1.26 × 10^−08^
	PU	6.00 × 10^−09^		1.26 × 10^−08^
**NMOR**	Rubber	2.99 × 10^−07^	6.70 × 10^+00^	2.00 × 10^−06^
	TPE	1.14 × 10^−07^		7.64 × 10^−07^
	TPU	1.20 × 10^−08^		8.04 × 10^−08^
	PU	4.60 × 10^−08^		3.08 × 10^−07^
**NDPhA**	Rubber	9.01 × 10^−08^	4.90 × 10^−03^	4.41 × 10^−10^
	TPE	5.70 × 10^−08^		2.79 × 10^−10^
	TPU	6.00 × 10^−09^		2.94 × 10^−11^
	PU	6.00 × 10^−09^		2.94 × 10^−11^
**NMEA**	Rubber	2.28 × 10^−07^	2.20 × 10^+01^	5.02 × 10^−06^
	TPE	2.28 × 10^−07^		5.02 × 10^−06^
	TPU	2.40 × 10^−08^		5.28 × 10^−07^
	PU	2.40 × 10^−08^		5.28 × 10^−07^

## Data Availability

Not applicable.
